# Incidence of laboratory-defined severe intravascular haemolysis across commercially available pulsed field ablation technologies for atrial fibrillation

**DOI:** 10.1093/europace/euaf185

**Published:** 2025-08-22

**Authors:** Stavros E Mountantonakis, Nicholas Beccarino, Humail Patel, Andres Castillo, Taha Siddiqui, Madhav Bhatt, Jonas Leavitt, Kristie M Coleman

**Affiliations:** Center for Arrhythmias, Northwell Cardiovascular Institute, 2000 Marcus Ave, Suite 300, New Hyde Park, NY 11042-1069, USA; Department of Electrophysiology, Lenox Hill Hospital, 100 East 77th Street, New York, NY 10075, USA; Center for Arrhythmias, Northwell Cardiovascular Institute, 2000 Marcus Ave, Suite 300, New Hyde Park, NY 11042-1069, USA; Center for Arrhythmias, Northwell Cardiovascular Institute, 2000 Marcus Ave, Suite 300, New Hyde Park, NY 11042-1069, USA; Center for Arrhythmias, Northwell Cardiovascular Institute, 2000 Marcus Ave, Suite 300, New Hyde Park, NY 11042-1069, USA; Center for Arrhythmias, Northwell Cardiovascular Institute, 2000 Marcus Ave, Suite 300, New Hyde Park, NY 11042-1069, USA; Center for Arrhythmias, Northwell Cardiovascular Institute, 2000 Marcus Ave, Suite 300, New Hyde Park, NY 11042-1069, USA; Center for Arrhythmias, Northwell Cardiovascular Institute, 2000 Marcus Ave, Suite 300, New Hyde Park, NY 11042-1069, USA; Department of Electrophysiology, Lenox Hill Hospital, 100 East 77th Street, New York, NY 10075, USA; Center for Arrhythmias, Northwell Cardiovascular Institute, 2000 Marcus Ave, Suite 300, New Hyde Park, NY 11042-1069, USA; Department of Electrophysiology, Lenox Hill Hospital, 100 East 77th Street, New York, NY 10075, USA

**Keywords:** Atrial fibrillation, Pulsed field ablation, Intravascular haemolysis, Clinical implementation

## Abstract

**Aims:**

Renal failure due to intravascular haemolysis (IH) has been reported after pulsed field ablation (PFA) of atrial fibrillation (AF). However, IH incidence using the accepted laboratory criteria is unknown.

**Methods and results:**

In this prospective observational study (Sept 2024–May 2025), consecutive patients undergoing PFA for AF with pentaspline (PS), circular array (CA), or lattice tip (LT) catheters were included. Pre- and post-procedural labs and haemolysis biomarkers were collected. Significant IH was defined as post-procedure free plasma haemoglobin > 100 mg/dL per haematology criteria. Logistic regression (pooled and stratified) was used to identify IH predictors. Among 245 patients (66.9 ± 10.6 years; 68.2% male; 48.2% persistent), PFA was performed using the LT (62), PS (108), or CA (75) catheters. There was a significant difference in the incidence of IH across technologies (37.0%, 26.1%, and 14.7% for PS, CA, and LT, *P* = 0.002). No demographic or clinical parameters were associated with higher IH risk, while the use of PS was the only independent predictor [odds ratio (OR) 3.42, *P* = 0.001] of IH. The number of PF lesions increased risk for IH only within the PS group (OR 1.03, *P* = 0.049). Routine post-ablation laboratories had poor sensitivity/specificity to define severe IH.

**Conclusion:**

Although over 17% of the cohort met the haematologic definition for significant IH, the absence of clinically significant renal impairment suggests that this threshold may not accurately reflect clinically meaningful haemolysis following PFA. The absence of clinical predictors or laboratory surrogates suggests that the rare risk of renal injury must be balanced with the well-established benefits of PFA when lesions are delivered in moderation optimizing tissue contact.

## Graphical Abstract

**Graphical Abstract:**
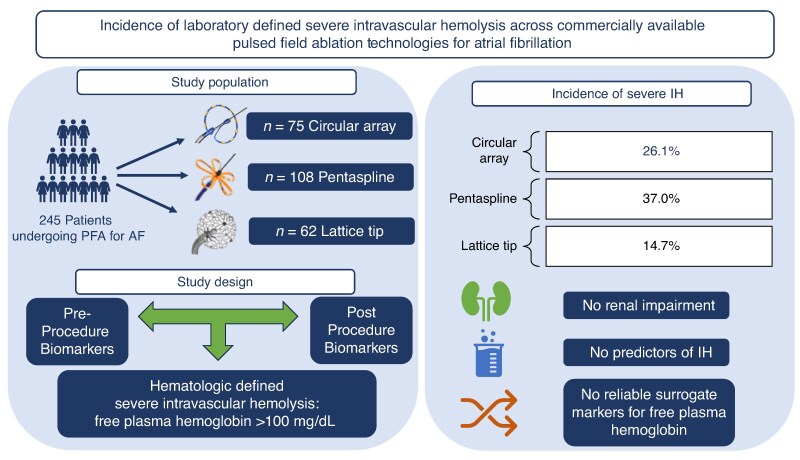

What’s new?

- High incidence of haematologic severe IH, but no renal impairment: 16.7% of patients experienced severe intravascular haemolysis (IH) as defined by haematologic criteria, varying by ablation technology used. Importantly, no clinically significant renal impairment was observed, suggesting that this threshold may not accurately reflect clinically meaningful haemolysis following PFA.
- No predictors of IH identified: The study failed to identify any independent clinical, procedural, or laboratory factors that predicted the occurrence of IH in the overall patient cohort.
- No reliable surrogate markers for free plasma haemoglobin (FPH) elevation: Commonly used markers like haptoglobin and LDH, as well as basic metabolic panel results, did not correlate with elevations in FPH, the most specific marker of IH.

## Introduction

Intravascular haemolysis (IH) has emerged as a potential complication of pulsed field ablation (PFA), a non-thermal modality increasingly utilized for catheter ablation of atrial fibrillation (AF).^1–3^ While early investigational device exemption (IDE) trials did not report IH, these studies lacked quantitative assessment and routine laboratory surveillance and were limited by stringent lesion delivery protocols.^4,5^ The realization that IH was a potential, albeit rare, complication for PFA was only actualized after real-world reports of clinically significant IH resulting in acute renal failure often requiring temporary dialysis.^6–8^ The incidence of IH is poorly defined given only severe cases resulting in renal injury are reported.^9,10^ Furthermore, it remains unclear if the incidence of IH differs across available PFA technologies.^11^

Subsequent studies have attempted to characterize IH employing various methodologies. However, variation in sampling time points, biomarker selection, clearance, and ablation technologies has led to inconclusive findings.^9–11^ Further ambiguity is introduced by the lack of a standardized definition of IH in the context of PFA, making interpretation and comparison across studies difficult.^12^ As a result of changes in fluid status during ablation and blood loss due to sheath management and haematomas, post-procedure haemoglobin may not be a reliable measure of IH.^13^ In addition, apoptosis due to PFA causes the release of non-specific indirect markers of cell destruction such as lactate dehydrogenase (LDH); therefore, a reliable indicator for IH remains undetermined.^12^ While free plasma haemoglobin (FPH) is a direct IH biomarker, it cannot be utilized as a routine screening laboratory index, due to the processing time required by a reference laboratory.^11^

### Objective

This study aims to determine the incidence, predictors (clinical, procedural, and laboratory), and surrogate markers of laboratory-defined severe IH, within a real-world PFA cohort across various commercially available technologies.

## Methods

### Study design and population

This is a prospective observational study of consecutive patients over the age of 18 undergoing PFA of AF with either the pentaspline (PS, Farawave, Boston Scientific), circular array (CA, PulseSelect, Medtronic), or lattice tip dual energy (LT, Affera Sphere-9, Medtronic) catheter between September 2024 and May 2025. All patients regardless of AF procedure type (paroxysmal, persistent, repeat) were included in the analysis. The PFA system utilized in the procedure, as well as the lesion set, was left to the discretion of the individual operator. This study received exempt approval from the Northwell Human Research Protection Program and a waiver of consent. The data that support the findings of this study are available from the corresponding author upon reasonable request.

### Ablation protocol

All procedures were performed under general anaesthesia. Anticoagulation was uninterrupted prior to ablation. Left atrial thrombus was ruled out pre-procedurally via cardiac CT or intra-procedurally using intracardiac echocardiography (ICE). Transseptal access was obtained using standard sheaths and guidance (fluoroscopy or ICE). Electroanatomical mapping systems (Carto, Ensite, Affera) were used to acquire left atrial anatomy. Unfractionated heparin was administered to maintain an activated clotting time (ACT) of ≥350 s. Each catheter was used according to the manufacturer’s guidelines and institutional protocols. Additional lesion sets including posterior wall isolation (PWI), mitral isthmus line (MIL), and cavotricuspid isthmus (CTI) ablation were performed based on operator judgment and arrhythmia inducibility. Radiofrequency energy was employed as needed, particularly for CTI ablation or completion of linear lesions. Intravenous fluids were not routinely administered to prevent haemolysis-related kidney injury.

### Blood sampling, biomarker assessment, and biochemical analysis

Blood samples were drawn at three time points: immediately prior to the procedure, immediately post-procedure, and on the morning of discharge (if not discharged the same day). The first 10 mL of blood was discarded for all samples. Samples were processed within 3 h using standardized laboratory techniques with the same assays across all participants. Free plasma haemoglobin was processed by a reference laboratory.

### Study endpoints

The primary endpoint was detection of clinically significant IH. To identify significant intravascular IH, severe IH was defined as greater than or equal to 100 mg/dL post-procedure as defined by haematology guidelines.^14^ Secondary outcomes included procedural parameters and incidence of acute kidney injury (AKI). Acute kidney injury was defined according to KDIGO guidelines: stage 1 as a ≥0.3 mg/dL increase or 1.5–1.9× baseline creatinine; Stage 2 as 2.0–2.9× baseline; and Stage 3 as ≥3.0× baseline, ≥4.0 mg/dL, or need for renal replacement therapy.

### Statistical analysis

Normally distributed data are presented as mean ± SD and non-normal data as median (IQR). Categorical data are presented as frequency (percentage of the total). Categorical variables were compared between PFA groups (pentaspline vs. circular array) using the Pearson *χ*² test or Fisher’s exact test. Continuous variables were compared using the independent samples *t*-test for normally distributed variables and the Mann–Whitney *U* test for non-normally distributed variables. Pairwise comparisons were performed using Bonferroni-adjusted *post hoc* tests following one-way ANOVA with equal variances assumed (see Supplementary material online, *Tables S1* and *S2*).

Univariate logistic regression analysis was performed to identify clinical (age, sex), procedural [total PF lesions, pulmonary vein isolation (PVI) only vs. PVI plus], and laboratory (pre-procedure GFR, creatinine, haemoglobin, haematocrit, RBC indices including MCH, MCV, MCHC and RBC, delta haemoglobin, delta creatinine, delta GFR), predictors of severe IH binarized as FPH > 100 mg/dL. Analyses were performed both overall and stratified by ablation catheter type (LT, PS, CA). Odds ratios (ORs) with 95% confidence intervals (CIs) are reported.

Spearman’s correlation analysis was also performed to assess the correlation between non-specific biomarkers of IH including LDH, bilirubin, aspartate aminotransferase (AST), alanine aminotransferase (ALT), alkaline phosphatase (ALP), haptoglobin, and laboratory-defined severe IH. The coefficient of determination (*R*²) and 95% CIs (calculated via Fisher’s z-transformation) were reported.

Analyses were stratified by catheter type to detect potential differences in predictors of IH among devices. All analyses were performed using Stata (v17; StataCorp LLC).

## Results

### Patient characteristics

A total of 245 patients (66.9 ± 10.6 years; 68.2% male, 48.2% persistent AF) undergoing PFA for AF during the study period were included in the analysis. The cohort had a mean age of 66.9 ± 10.6 years, 68.2% were male, and 48.2% presented with persistent AF. The mean CHA_2_DV_2_ASc score was 2.44 ± 1.52. Hypertension (56.7%), hyperlipidaemia (42.0%), and current or former tobacco use (35.9%) were the most prevalent comorbidities. There were no significant differences in left ventricular ejection fraction or left atrial volume index between ablation technologies. Baseline characteristics for the study population are displayed in *Table 1*.

**Table 1:** Baseline characteristics of study cohort by ablation technology

	Lattice tip (*n* = 62)	Pentaspline (*n* = 75)	Circular array (*n* = 108)	*P*-value
Age, mean (SD)	67.69 (9.76)	66.86 (10.54)	67.00 (11.56)	0.877
Gender (male %)	69.4%	66.7%	70.7%	0.839
BMI, mean (SD)	30.52 (9.53)	29.75 (6.25)	27.76 (5.19)	0.082
HTN (%)	66.7%	65.4%	44.0%	**0**.**006**
Heart failure (%)	20.0%	25.2%	22.7%	0.739
Coronary stent (%)	11.7%	11.2%	8.0%	0.724
Myocardial infarction (%)	3.3%	3.7%	5.3%	0.814
CAD (%)	30.0%	25.2%	24.0%	0.709
Prior cardiac surgery (%)	11.7%	9.3%	9.3%	0.872
PAD (%)	10.0%	3.7%	2.7%	0.108
Cardiomyopathy (%)	15.3%	20.6%	20.0%	0.687
Pulmonary disease (%)	20.3%	15.0%	10.7%	0.297
DM (%)	22.0%	15.0%	14.7%	0.434
Renal disease (GFR < 50) (%)	1.7%	7.5%	6.7%	0.291
Stroke (%)	8.5%	7.5%	4.0%	0.526
Anaemia (%)	5.0%	8.5%	4.2%	0.452
EF (%), mean (SD)	56.41 (9.20)	54.40 (12.96)	55.66 (11.08)	0.589
LAVI (mL/m²), mean (SD)	41.83 (19.31)	36.34 (14.96)	41.07 (20.68)	0.426

Bold values indicate *P* values <0.05.BMI, body mass index; HTN, hypertension; CAD, coronary artery disease; PAD, peripheral artery disease; DM, diabetes mellitus; EF, ejection fraction; LAVI, left ventricular ejection fraction.

### Procedural characteristics

All patients in the study population underwent ablation with PFA, using the lattice tip dual energy (LT, *n* = 62), circular array (CA, *n* = 108), or pentaspline (PS, *n* = 75) catheters (*Table 2*; Supplementary material online, *Table S1*). Redo ablation procedures were more likely to be performed with the LT (12.9%) or CA (12.0%) catheters compared to the PS (2.8%). Pulmonary vein isolation (PVI) was more frequent with the PS catheter (60, 55.6%), compared to patients receiving ablation with the CA (21, 28.0%) and LT (14, 22.6%) catheters. The mean number of total PFA applications was significantly higher in the LT group compared to the CA and PS (LT: 71.8 ± 33.9, CA: 50.1 ± 14.3, PS: 56.9 ± 19.2, *P* < 0.001). The PVI-only PFA applications showed a similar trend (LT: 57.4 ± 31.6, CA: 44.5 ± 17.3, PS: 44.2 ± 13.5). Total procedure time was the shortest with the LT catheter (111.8 min ± 30.4), while comparable between CA (125.2 min ± 44.0) and PS (127.6 min ± 41.3). Fluoroscopy time was longest in the PS group (19.3 min ± 12.6), while comparable between the CA (9.5 min ± 12.6) and LT (8.7 min ± 11.4) groups.

**Table 2:** Procedural characteristics

	Lattice tip	Pentaspline	Circular array	*P*-value
Total heparin dose (IU), mean (SD)	20 890 (5265)	19 130 (6318)	19 908 (4853)	0.191
Max ACTs, mean (SD)	388.08 (44.40)	360.59 (47.05)	378.71 (50.64)	**0**.**002**
Total volume in (mL), mean (SD)	1400 (376.33)	1278.5 (330.08)	1128.87 (325.98)	**<0**.**001**
Complication^a^ (%)	0 (0.0%)	5 (4.6%)	0 (0.0%)	0.053
PVI PF applications	57.34 (31.65)	44.17 (13.54)	44.49 (17.30)	**<0**.**001**
PWI (%)	38 (61.3%)	36 (33.6%)	46 (61.3%)	**<0**.**001**
CTI (%)	26 (42.6%)	14 (13.0%)	30 (40.0%)	**<0**.**001**
MIL (%)	13 (22.8%)	3 (2.8%)	5 (6.8%)	**<0**.**001**
PVI only (%)	14 (22.6%)	60 (55.6%)	21 (28.0%)	**<0**.**001**
Total PF applications, mean (SD)	71.84 (33.91)	50.13 (14.28)	56.88 (19.25)	**<0**.**001**
Total fluoroscopy time (min), mean (SD)	8.68 (11.43)	19.31 (12.56)	9.46 (12.55)	**<0**.**001**
Total procedural time (min), mean (SD)	111.85 (30.36)	127.62 (41.34)	125.16 (43.97)	0.061
Length of stay (days), mean (SD)	0.63 (0.49)	0.74 (0.65)	0.46 (0.58)	**0**.**011**

Bold values indicate *P* values <0.05.ACT, activated coagulation time; PVI, pulmonary vein isolation; CTI, cavotricuspid isthmus line; MIL, mitral isthmus line; PF, pulsed field.

^a^Five complications noted in the pentaspline group were related to groin access haematoma.

Posterior wall isolation (LT: 61.3%, PS: 33.6%, CA: 61.3%, *P* < 0.001) and mitral isthmus lines (LT: 22.8%, PS: 2.8%, CA: 6.8%, *P* < 0.001) were also more frequently applied in the LT and PS cohorts. Cavotricuspid isthmus ablation was most frequently performed with the LT catheter (26, 42.6%). Nearly half of the patients in the CA group received CTI (30, 40.0%), with the CA catheter being utilized for the majority (25 patients). While 14 patients in the PS group received CTI, RF was utilized for the CTI line in nine patients.

In the pentaspline group, five vascular access complications were noted (4.6%). Length of stay was marginally longer in the PS group 0.74 ± 0.65 days compared to the LT (0.63 ± 0.49) and CA (0.46 ± 0.58) groups (*P* = 0.011).

### Intravascular haemolysis biomarkers deltas by ablation technology

**Figure 1:**
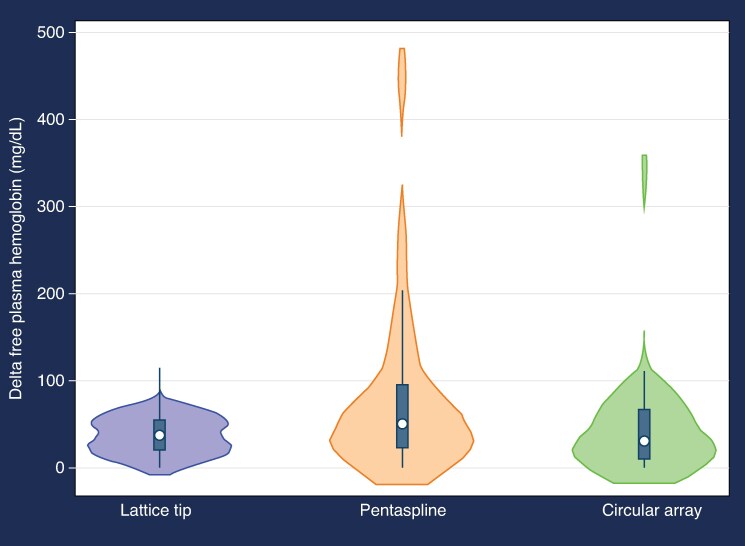
Change in free plasma haemoglobin stratified by catheter type.

Pre- to post-procedural changes in laboratory biomarkers varied across ablation technologies (*Table 3* and *Figure 1*; Supplementary material online, *Table S2*). Patients treated with the PS catheter experienced significantly greater increases in delta FPH [median 50.4 mg/dL (IQR 22.1–95.8)] compared to those treated with LT [30.4 mg/dL (IQR 9.0–67.6)] or CA [36.8 mg/dL (IQR 19.4–55.9); *P* < 0.001]. Compared to the CA, the PS was associated with a significantly greater increase in FPH (delta FPH +41.5 mg/dL, *P* = 0.002), while the LT demonstrated a significantly lower decrease in FPH (delta FPH −35.9 mg/dL, *P* = 0.019) compared to the PS. Patients who underwent ablation with the PS catheter had significantly greater reductions in red blood cell (RBC) count [median 0.505 million/µL (IQR 0.35–0.66); *P* = 0.003] and haematocrit [median 5.0% (IQR 3.5–6.2); *P* = 0.002] compared to the other technologies. Significant differences were also seen with alanine aminotransferase and alkaline phosphatase.

**Table 3:** Deltas for pre- and post-procedure biomarkers by ablation technology

	Lattice tip [median (IQR)]	Pentaspline [median (IQR)]	Circular array [median (IQR)]	*P*-value
delta_FPH	30.4 [9.0–67.6]	50.4 [22.1–95.8]	36.8 [19.4–55.9]	**<0**.**001**
delta_LDH	24 [9.0–40.0]	21 [6.0–46.0]	22 [13.0–36.0]	0.857
delta_Hapt	22.5 [13.5–31.5]	27 [20.0–32.0]	27 [20.0–36.0]	0.242
delta_HGB	1.9 [1.3–2.25]	1.6 [1.1–1.9]	1.4 [1–1.9]	0.316
delta_Bili	0.1 [0–0.3]	0.2 [0.1–0.5]	0.1 [0.1–0.3]	0.169
delta_Creat	0.065 [0.04–0.13]	0.06 [0.03–0.1]	0.05 [0.02–0.09]	0.124
delta_RBC	0.69 [0.46–0.76]	0.505 [0.35–0.66]	0.52 [0.3–0.64]	**0**.**003**
delta_HCT	6.5 [4.8–7.3]	5 [3.5–6.2]	4.7 [2.8–6.2]	**0**.**002**
delta_GFR	5.0 [2.0–8.0]	3.0 [2.0–7.0]	4.0[2.0–8.0]	0.200
delta_AST	6 [2.0–13.0]	3.5 [2.0–17.0]	5 [3.0–12.0]	0.884
delta_ALT	4.0 [2.0–6.0]	3.0[2.0–5.0]	3.0[2.0–4.0]	**0**.**014**
delta_ALP	16[10.5–21.0]	10.0[7.0–15.0]	10.5[6.0–13.0]	**<0**.**001**

Bold values indicate *P* values <0.05.FPH, free plasma haemoglobin; LDH, lactate dehydrogenase; Hapt, haptoglobin; HGB, haemoglobin; Bili, bilirubin; Creat, creatinine; RBC, red blood cell count; HCT, haematocrit; GFR, glomerular filtration rate; AST, aspartate aminotransferase; ALT, alanine aminotransferase; ALP, alkaline phosphatase.

### Severe intravascular haemolysis and change in red blood cell indices

There were no statistically significant differences in RBC indices between patients with IH compared to those without IH in the overall cohort and in stratified analysis.

### Clinical, procedural, and laboratory predictors of IH

**Figure 2:**
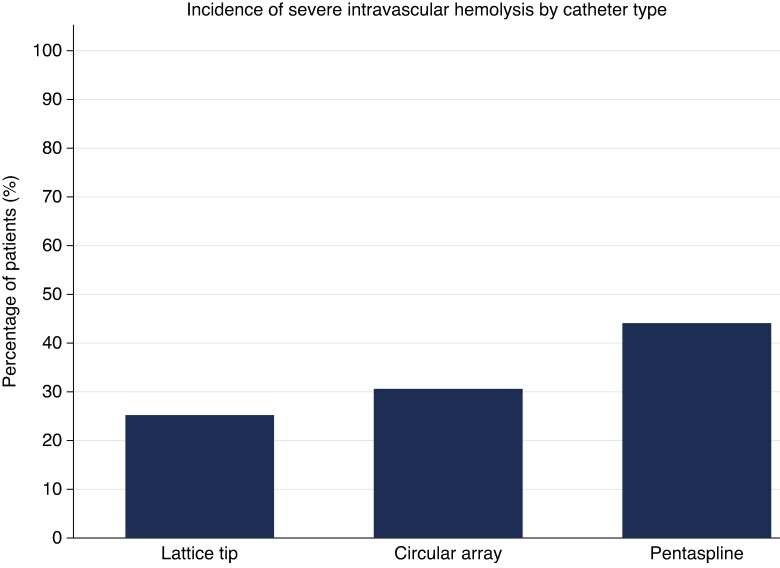
Incidence of severe intravascular haemolysis by pulsed field ablation technology.

Of the 245 patients, the overall incidence of haematologic-defined severe IH (FPH ≥ 100 mg/dL) was 16.7% (41/245). There was a significant difference in the incidence of IH among technologies used (37.0%, 26.1%, 14.7% for PS, CA and LT catheter respectively, *P* = 0.002) (*Figure 2*).

In univariate logistic regression analysis, after adjusting for age, sex, baseline GFR, creatinine, haemoglobin, haematocrit, RBC indices (MCH, MCV, MCHC, RBC), and ablation extent (PVI only vs. PVI plus), none of these variables significantly predicted severe IH overall. There was a significant association between IH and the type of ablation technology used (*P* = 0.002), with PS (OR 3.42, 95% CI 1.62–7.24) having a significantly greater risk of IH compared to the LT reference group. Within the pentaspline group, the total number of PFA applications was a significant predictor of IH (OR 1.03, 95% CI 1.00–1.06, *P* = 0.049). Across the total cohort and stratified by ablation technology, no statistically significant relationship was found between delta haemoglobin, delta creatinine, delta GFR, post-procedure haemoglobin, post-procedure GFR or post-procedure creatinine, and IH.

### Correlation between non-specific biomarkers and laboratory-defined severe intravascular haemolysis

Spearman’s correlation analysis performed on the pooled dataset showed no significant correlation between IH and LDH, bilirubin, AST, ALT, ALP, or haptoglobin, whether considering the pre-procedure, post-procedure values, or deltas. In the PS group, statistically significant correlations were found between IH and post-procedure ALT, pre- and post-procedure ALP, and delta ALP. No such correlations were observed in the LT or CA groups. No reliable surrogate indicator of severe IH was identified among non-specific biomarkers or common laboratory values.

## Discussion

Through this large, prospective analysis, we report that (i) the overall incidence of PFA-induced haematologic-defined severe IH was 16.7% and differed by technology, although no clinically significant renal impairment was observed; (ii) no independent clinical, procedural, or laboratory predictors of IH were identified for the overall cohort; and (iii) no surrogate markers that correlated with FPH elevators were delineated.

### Incidence of pulsed field ablation–induced intravascular haemolysis

The last 25 years have marked a tremendous period of advancement in the physiologic understanding of AF as a progressive disease, the advent of an interventional procedure based on a novel observation, as well as the exponential innovations in the technologies used to perform the procedure.^14,15^ Pulsed field catheter ablation technology represents the pinnacle achievement of a decades long undertaking to deliver an efficacious, safe, and efficient tool to optimize clinical outcomes for patients with AF.^16^ Similarly to early radiofrequency catheter ablation studies that reported a low rate of asymptomatic acute emboli with unclear clinical significance on cerebral magnetic resonance imaging post-ablation,^17^ with the rapid widespread adoption of PFA into standard clinical practice, the risk of IH has emerged as a potential complication also with unclear clinical signfigance.^6^

Early reports of PFA-induced IH, often requiring temporary dialysis to mitigate, were alarming for a technology heralded for its safety profile.^6–8^ Further investigation is therefore needed before generalizing the low (0.03%) incidence of PFA-induced renal failure to the broader population, mirroring the experience with silent cerebral embolic where the observed frequency did not correlate with clinical sequelae.^6,14^ Over the last year, several studies have been conducted to ascertain the true incidence rate of IH compared, most frequently, to radiofrequency ablation.^9–12,18^ These early reports showed the incidence of IH was rare, albeit poorly defined.^19,20^ Since IH is a new phenomenon in the EP literature, in this study, we elected to follow the official haematologic definition to offer deeper insight into how RBCs respond to PFA and to compare the differential effects across technologies. While the current literature equates renal failure as a surrogate of haemolysis, renal failure is in fact an effect of haemolysis and largely affected by renal function reserves. The seemingly rare reports of PFA-induced renal failure appear to be impacted by a multitude of confounders that are not adjusted for. Free plasma haemoglobin is an upstream biomarker in the haemolytic process and its sequelae and therefore the most sensitive marker of its occurrence.

### Laboratory surrogates of severe intravascular haemolysis

Haptoglobin and LDH have been reported as indirect biomarkers of IH,^9–12^ while direct biomarker FPH has been evaluated in a subset of patients undergoing PFA.^11,18^ The clinical utility of haptoglobin, LDH, or FPH as a screening laboratory index is yet to be determined.^18^ These labs are not assessed in routine clinical workflows, and peak level of these biomarkers is frequently reported to be >24 h post-procedure. In the present analysis, ‘severe IH’ was defined as >100 mg/dL per the haematologic literature, and although nearly a fifth of the cohort experienced the primary outcome, no cases of significant procedure-related renal dysfunction were observed. Free plasma haemoglobin is thought to be the most specific and sensitive direct biomarker of IH; however, processing is required by a reference laboratory and routinely results in 24–48 h.^11^ Therefore, the optimal time to uniformly assess post-procedure biomarkers remains a challenge that must balance pathophysiology with practice clinical considerations, such as standard discharge timelines.^21^ While in the haematologic literature FPH is useful for predicting complications in situations involving sustained exposure to IH, as in the case of sickle cell disease or extracorporeal membrane oxygenation (ECMO), whether this screening modality is relevant to acute cardiac exposure is inconclusive.^22^ The coronary artery bypass graft surgery literature provides valuable clinical equipoise for assessing the risk of transient acute elevations in FPH. Wetz *et al*.^23^ followed a similar protocol to the present study for evaluating pre- and post-procedure biomarkers and found no correlation between increased FPH, decreased haptoglobin, and renal impairment. In the present analysis, changes in routine labs including LDH, haptoglobin, haemoglobin, bilirubin, creatinine, GFR, and AST across the three ablation modalities did not correlate with FPH defined IH. The observed reductions in RBC count and haematocrit in the PS group are likely a consequence of blood loss due to sheath management, which is notably larger with the PS catheter (12 French vs. CA; 9 French, LT: 8 French).^16^

### Predictors of pulsed field ablation–induced intravascular haemolysis

Intravascular haemolysis has also previously been highlighted in the cardiovascular procedural landscape as a serious, but rare complication following implantation of prosthetic heart valves.^24^ Similar to the present analysis, clinical, procedural, or laboratory predictors associated with IH could not be identified. Rather, IH was found to be associated with prosthesis related variables.^25^ In the present analysis, the only independent predictor of IH in the overall cohort was the use of the PS catheter, while the total number of PF applications was significant when assessing the PS subgroup. However, given the different architecture of the PFA delivery systems, it is arbitrary to attempt to assign a lesion threshold as a cut-off for PFA-induced IH and animal models have shown different dose dependency varies by catheter type.^7,19,20,26^ Animal studies have shown that tissue contact is important not only for lesion depth but also for the inadvertent energy dispersion to the blood flow.^19^

Similar to the first-generation prosthetic valves, a distinct mechanism for PFA-induced IH which could be attributable to catheter design, waveform, and or number of applications delivered.^19,27^ The mechanism inducing shear stress in the CABG literature is well reported; however, further catheter-specific translational studies are needed to determine the mechanism by which PFA induces RBC sub-lethal or lethal injury resulting in the release of potentially nephrotoxic FPH.^22,23^ The phenotype and clinical presentation of haemolysis is highly variable and impacted by patient specific clinical covariates. The sequelae of haemolysis could potentially be delayed, and damaged RBCs may continue to circulate for hours or days before rupture potentially causes renal injury or anaemia; however, this protocol was not designed to assess for delayed effects of PFA.

### Clinical implications

Current clinical evidence, largely comprised of small heterogeneous cohorts with variable methodologies for defining and evaluating PFA-induced haemolysis, warrants cautious interpretation.^26^ This large, prospective study, employing a rigorous protocol for evaluating the clinical significance of PFA-induced haemolysis using the haematologic definition, suggests that transient FPH elevations are not associated with significant renal impairment. Furthermore, the study found no correlation between transient FPH elevation and biomarkers of haemolysis (LDH, haptoglobin) or basic metabolic panel surrogates. These findings indicate that the risk of clinical haemolysis is extremely low across technologies and attenuated by the well-established benefits of PFA when lesions are delivered in moderation with a focus on ensuring tissue contact. With any novel technology that is introduced into widespread clinical practice, ongoing post-market approval registries for PFA catheters are designed to surveil for clinically significant risks previously not observed during the pivotal trials (NCT04198701, NCT04524364, NCT06431815) in combination with mechanistic translation studies, which will determine whether the perceived risk of PFA-induced IH aligns with the actual clinical impact.

### Limitations

Given the observational nature of this study, there are inherent limitations. Despite including consecutive patients undergoing PFA for AF with ablation system selection at the operator’s discretion, the cohort was well balanced in terms of clinical characteristics. The circular array and lattice tip catheters were used for more PVI plus and repeat procedures. Consistent with standard clinical practice, operators demonstrated a preference for specific catheters based on their ablation experience; however, there was no statistically significant association between a specific operator and elevated FPH level per catheter type. While inter-operator variability could have been introduced by catheter selection being left to operator discretion, it likely reduces intra-operator variability potentially enhancing procedural consistency at the operator level. Methodologically, we elected to collect samples immediately post-procedure as FPH clearance is thought to occur by 24 h.^11^ The timing of sampling immediately post-procedure might not have recorded the peak values of all haemolysis biomarkers; however, it enabled standardization of sampling that allowed comparison between the three groups, while following a protocol that is feasible in current clinical practice. Due to the frequency of same day discharge as routine practice, 24 h post-procedure laboratory values were only available for analysis in a subset of the cohort. To determine the cut-off value needed to achieve a clinically significant change in FPH, we elected to use the definition of severe IH established by haematology guidelines, although further research is needed to validate a meaningful threshold. We acknowledge that not all cases of laboratory-defined severe IH had clinically apparent sequelae; however, there is a clear need in the EP community for an objective quantification of PFA-related haemolysis.

## Conclusions

Although over 17% of the cohort met the haematologic definition for significant IH, the absence of clinically significant renal impairment suggests that this threshold may not accurately reflect clinically meaningful haemolysis following PFA. The absence of consistent clinical predictors or laboratory surrogates of elevated FPH suggests that the rare risk of renal injury must be balanced with the well-established benefits of pulsed field technology for catheter ablation of AF when lesions are delivered in moderation optimizing tissue contact.

## Supplementary Material

euaf185_Supplementary_Data

## Data Availability

Data will be made available on request to the corresponding author.
